# Evaluating the Effectiveness of NoteAid in a Community Hospital Setting: Randomized Trial of Electronic Health Record Note Comprehension Interventions With Patients

**DOI:** 10.2196/26354

**Published:** 2021-05-13

**Authors:** John P Lalor, Wen Hu, Matthew Tran, Hao Wu, Kathleen M Mazor, Hong Yu

**Affiliations:** 1 Department of Information Technology, Analytics, and Operations Mendoza College of Business University of Notre Dame Notre Dame, IN United States; 2 Department of Computer Science University of Massachusetts Lowell Lowell, MA United States; 3 Department of Psychology and Human Development Peabody College Vanderbilt University Nashville, TN United States; 4 Meyers Primary Care Institute University of Massachusetts Medical School/Reliant Medical Group/Fallon Health Worcester, MA United States; 5 Department of Medicine University of Massachusetts Medical School Worcester, MA United States; 6 College of Information and Computer Sciences University of Massachusetts Amherst Amherst, MA United States; 7 Center for Healthcare Organization and Implementation Research Bedford Veterans Affairs Medical Center Bedford, MA United States

**Keywords:** health literacy, crowdsourcing, natural language processing, information storage and retrieval, psychometrics, intervention, literacy, electronic health record, efficacy, comprehension

## Abstract

**Background:**

Interventions to define medical jargon have been shown to improve electronic health record (EHR) note comprehension among crowdsourced participants on Amazon Mechanical Turk (AMT). However, AMT participants may not be representative of the general population or patients who are most at-risk for low health literacy.

**Objective:**

In this work, we assessed the efficacy of an intervention (NoteAid) for EHR note comprehension among participants in a community hospital setting.

**Methods:**

Participants were recruited from Lowell General Hospital (LGH), a community hospital in Massachusetts, to take the ComprehENotes test, a web-based test of EHR note comprehension. Participants were randomly assigned to control (n=85) or intervention (n=89) groups to take the test without or with NoteAid, respectively. For comparison, we used a sample of 200 participants recruited from AMT to take the ComprehENotes test (100 in the control group and 100 in the intervention group).

**Results:**

A total of 174 participants were recruited from LGH, and 200 participants were recruited from AMT. Participants in both intervention groups (community hospital and AMT) scored significantly higher than participants in the control groups (*P*<.001). The average score for the community hospital participants was significantly lower than the average score for the AMT participants (*P*<.001), consistent with the lower education levels in the community hospital sample. Education level had a significant effect on scores for the community hospital participants (*P*<.001).

**Conclusions:**

Use of NoteAid was associated with significantly improved EHR note comprehension in both community hospital and AMT samples. Our results demonstrate the generalizability of ComprehENotes as a test of EHR note comprehension and the effectiveness of NoteAid for improving EHR note comprehension.

## Introduction

Access to and demand for heath information has led to a greater focus on patient-centered care [1,2]. Patient-centered care “makes the patient and their loved ones an integral part of the care team who collaborate with health care professionals in making clinical decisions” [2]. While prior work has shown that more active involvement by patients can lead to better outcomes [3,4] and that patients are more proactive than ever in seeking out health information [5,6], challenges remain for patients with low health literacy. The National Assessment of Adult Literacy estimates that approximately 36% of Americans have health literacy levels rated as “basic” or “below basic” [7]. This estimate, combined with the finding that physicians often overestimate the health literacy of their patients [8,9], shows that there is a gap between patient desire for health information and their ability to understand it. This gap can lead to adverse effects for patients as well as higher costs for health care centers [10-16]. Identifying low health literacy individuals and providing resources to improve their understanding are two key areas in population health research [17-20].

One aspect of health literacy that has become more critical in recent years is eHealth literacy [21,22]. As increasing numbers of patients are able to view their medical records online via patient portals (eg, the OpenNotes project) [23], there is a growing need for eHealth literacy tests and interventions to assess and improve eHealth literacy. One tool for assessing eHealth literacy is the ComprehENotes test [24], which tests the ability of individuals to understand free-text notes in a sample of electronic health records (EHRs). The ComprehENotes test consists of multiple-choice questions generated by groups of physicians and medical researchers, and validated using item response theory (IRT). Research has shown that providing access to NoteAid, an educational intervention tool that automatically defines medical terms in lay terms [18,25], improves scores on ComprehENotes items [26]. However, participants in these studies were recruited from the Amazon Mechanical Turk (AMT) platform, and thus may not represent the typical patient population [27,28]. For example, the AMT participants’ self-reported demographic characteristics revealed that these participants tended to be younger and better educated than patients at risk of low health literacy [7].

This study examined the impact of NoteAid on participants recruited from Lowell General Hospital (LGH), a community hospital in Massachusetts, including diabetes patients and their friends and family members. We sought to answer the following research question: “Does NoteAid improve EHR note comprehension for participants recruited from a community hospital?” (RQ1).

As a secondary goal, we sought to analyze the differences in performance between participants at the community hospital and participants on the AMT platform. Prior work has shown that NoteAid leads to improved scores on the ComprehENotes test for AMT participants [26]. However, overall scores for all participants on AMT were relatively high, and the population of AMT participants did not include groups typically at higher risk for low health literacy. Therefore, we compared performance on ComprehENotes between participants recruited from a community hospital and participants recruited from AMT to identify differences. The second research question was as follows: “Are participants at a community hospital different from AMT participants in terms of their EHR note comprehension levels as measured by the ComprehENotes test?” (RQ2).

As a third goal, we sought to determine whether NoteAid is equally effective for improving EHR note comprehension between participants at a community hospital and participants on the AMT platform. The third research question was as follows: “Is NoteAid equally effective or differentially effective for community hospital participants as for AMT participants in improving EHR note comprehension?” (RQ3).

Finally, we investigated the performance of different demographic groups on the ComprehENotes test, both with and without NoteAid, to see if ComprehENotes scores vary across subgroups, and whether NoteAid is equally effective across these subgroups. The fourth and fifth research questions were as follows: “Is EHR note comprehension consistent across different demographic groups?” (RQ4) and “Is NoteAid equally effective or differentially effective across different demographic groups?” (RQ5).

## Methods

### Overview

The work in this study was approved by the Institutional Review Boards (IRBs) at the University of Massachusetts Medical School and LGH. All participants from LGH were shown an information sheet describing the study, had the ability to ask questions before participating, and provided verbal informed consent before participating. AMT participants provided electronic informed consent before participating.

### NoteAid

**Figure 1:**
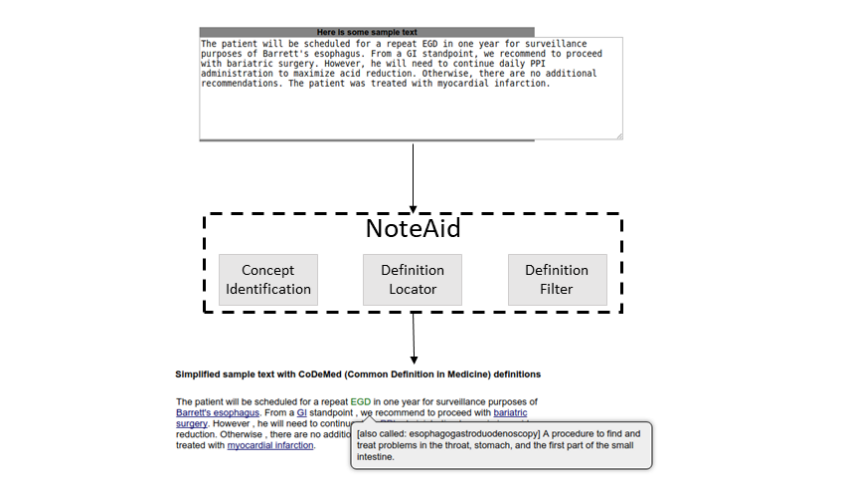
An example of medical terminology definition using NoteAid (image source: [26]).

NoteAid is a web-based natural language processing (NLP) system for linking medical jargon to lay-language definitions [18,25]. The following two components are central to NoteAid: a repository of lay definitions for medical terms (CoDeMed) and the NLP system for linking medical concepts to these definitions (MedLink). NoteAid is implemented as a web application, where users can navigate to the NoteAid website and enter a snippet of text from their own EHR note. NoteAid will then process the note text and display the note with terms defined via tooltip text. Defined terms are underlined, and users can display a term definition by moving the mouse cursor over the term (Figure 1).

### ComprehENotes

#### Overview

**Figure 2:**
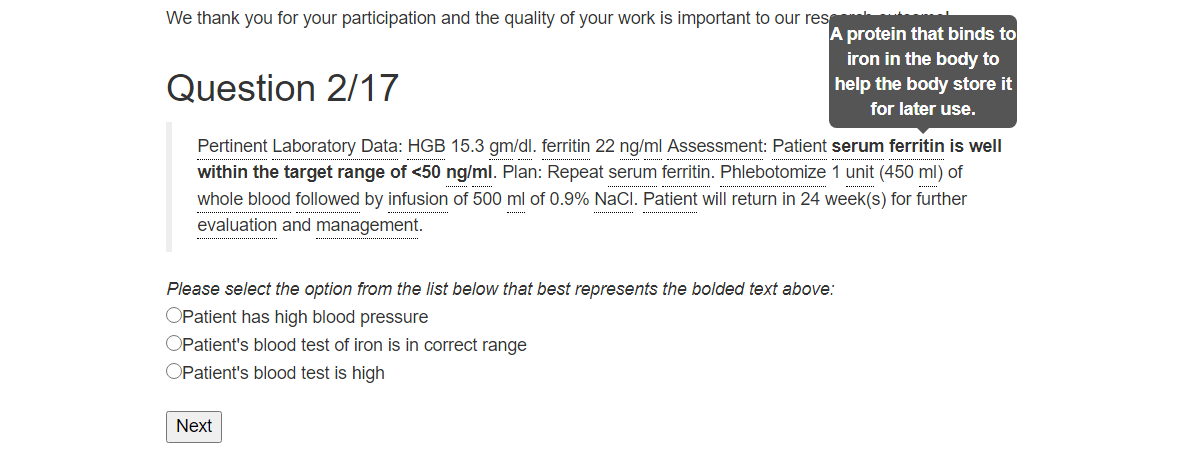
An example of a ComprehENotes test question with embedded NoteAid definitions as implemented on the web application. In this example, the definition of “ferritin” (gray box) is useful in understanding that the bold text describes a blood iron test.

The ComprehENotes test is the first test to directly assess the ability of individuals to comprehend EHR notes. The ComprehENotes test consists of 14 passages taken from deidentified EHR notes. A section of the passage is presented in boldface, and the test takers are asked to select which of three options is the closest in meaning to the bold text (Figure 2). As detailed by Lalor et al [24], the ComprehENotes test was built by a group of physicians and nonclinical medical researchers using the sentence verification technique [29]. Questions were administered to a sample of 660 English-speaking adults on AMT. The psychometric properties of the questions were subsequently analyzed using the IRT method to confirm the performance of the test questions [24,30].

Prior work [24] has shown that ComprehENotes test scores are consistent with demographic expectations with regard to health literacy (ie, less educated respondents score lower than more educated respondents). In addition, providing access to lay definitions for medical terms via NoteAid is associated with higher scores on ComprehENotes [26].

#### ComprehENotes Administration at a Community Hospital

For the community hospital participants, we implemented the ComprehENotes test as a web application on a tablet, which allowed for flexibility in terms of delivery and intervention modifications. The hardware used was one Microsoft Surface Pro and one Apple iPad Pro 11. When a participant loaded the application, he or she was randomly assigned to either the control or intervention group. Participants provided demographic information on the app, and then proceeded to take the test. The test was administered one question at a time. The order of the questions was randomized. Responses were directly collected and stored on our server. Other than self-reported demographic information, no user information was stored on the server.

#### ComprehENotes Administration on AMT

For AMT participants, the ComprehENotes test was implemented as a web application where quality control questions were additionally included to ensure that participants completed the task to the best of their ability. Specifically, the AMT participants were given three quality control “check questions” interspersed throughout the ComprehENotes test. These questions were included to ensure that the AMT participants were paying attention as they were completing the task [31]. If participants answered a check question incorrectly, they were presented with a message indicating that they have answered a check question incorrectly, and were given the option to start the task over or exit the window without completing the task.

#### Integrating NoteAid With ComprehENotes

For the intervention groups (community hospital and AMT), each ComprehENotes question was preprocessed by NoteAid and the results were embedded into the test web application directly. Definitions for terms were added to the web application as tooltip text. Terms were underlined to indicate that a definition was available, and when a participant hovered over a defined term, the definition would automatically display (Figure 2). This behavior was also described in the introductory text paragraph of the web application so that the participants were aware of the definitions and knew how to access them.

### Data Collection

#### Community Participant Recruitment

With IRB approval, participant recruitment was conducted at LGH. Staff members of the research team (WH and MT) approached diabetes patients in the waiting room before or after their appointments. Patients and persons accompanying patients in the waiting room were eligible to participate if they were over 18 years old, able to speak and read English, and comfortable using a tablet. In some cases, patients who started the survey could not finish it before their appointments and therefore were not included in the study. Partial responses were not included in our analyses.

The staff approached a potential participant and asked whether he/she would be interested in a short online English survey to see how he/she understands medical jargon terms in doctors’ notes. Then, the staff explained the fact sheet, including information regarding time to complete, IRB approval, and contact information. The staff also noted that the EHR notes in the questionnaire were not from their own personal EHR notes. We informed participants that once the survey started, they could discontinue participation at any time. Each participant was given a US $10 gift certificate. Participants were randomly assigned to the control or treatment group when they accessed the web page to complete the test via a random number generator implemented in Python.

#### AMT Participant Recruitment

A total of 200 participants were recruited on AMT (100 in the control group and 100 in the intervention group). Task visibility on the AMT platform was restricted to AMT workers located in the United States with prior task approval rates above 95%. The prior task approval rate percentage was used as an indicator of high-quality prior work, and selecting the United States as the location was used as a proxy for English proficiency. We did not collect any medical history information from participants. While the test administered to the AMT and community hospital participants was the same, there were several differences in test administration. First, AMT participants completed the task remotely, while the community hospital participants completed the task locally and under the supervision of research staff. Second, community hospital participants were randomly assigned to either the control or intervention group upon enrollment. On AMT, the task was implemented as a parallel randomized study. We first collected responses from 100 AMT participants for the control task (ie, no access to NoteAid). We then created and released a second AMT task with the intervention task. This intervention task was not available to AMT participants who participated in the control task. AMT participants were paid US $3 to complete the task. In pilot studies, we observed that AMT participants typically took between 15 and 20 minutes to complete the task; therefore, a US $3 payment approximated a US $9 to $12 hourly wage.

### Data Analysis

For our specific hypotheses regarding the effects of NoteAid in the two participant recruitment sources (hereafter referred to as “*source*”) (RQ1-3), we ran a two-way analysis of variance (ANOVA) to compare the four groups in our data set, using the proportion of the passage-item pairs answered correctly as the dependent variable and *source* (community hospital vs AMT) and *condition* (control vs intervention) as two crossed factors. Specifically, for our third research question, we tested the interaction effect between *source* and *condition*. An interaction would indicate that NoteAid’s effect on the test score differs between AMT participants and community hospital participants. In this case, for our primary research question, we would compare community hospital participants in the control and intervention groups to determine the effectiveness of NoteAid among community hospital participants, and for our secondary research question, we would compare the community hospital participants and AMT participants separately under the intervention and control conditions. If the interaction is not significant, the two main effects would be tested to address the primary and secondary research questions.

To examine the effects of participant demographic characteristics and possible variations in the effect of NoteAid on the test score among different demographic categories (RQ4-5), we further considered a model with three-way interactions among *condition*, *source*, and each of the demographic variables (age, race, education, and gender) along with relevant lower order effects. The four effects concerning the same demographic variables were treated as one family, and each of them was tested at level α’=.0125. If an interaction was detected and simple effects were examined, their tests were further adjusted with Bonferroni correction. Pairwise comparisons among demographic categories were also adjusted with the Bonferroni method.

Given the discrete nature of the dependent variable and the likely ceiling effect due to the overall good performance, we supplemented the ANOVA with generalized linear models (GLMs), treating the dependent variable as a binomial outcome with possible overdispersion to account for individual differences.

## Results

### Demographics

We recruited a total of 188 participants at the community hospital location from the end of December 2019 to the beginning of March 2020. Results from 174 participants were included in the final analysis. Of the 174 participants, 141 were patients and 33 were persons accompanying patients. Fourteen participants were recruited and began the task, but did not complete it as they were called to their appointments and therefore were not included in our final analyses.

Characteristics of the AMT participants and the community hospital participants are presented in Table 1. The distribution of age was very different between the groups. The AMT participants were primarily younger, with the majority of AMT participants under 34 years old, while the majority of community hospital participants were over 55 years old. There were also differences in education. A majority of AMT participants had a bachelor’s or master’s degree, while fewer community hospital participants had either degree. The majority of community hospital participants had at the most an associate’s degree.

Within the community hospital sample, the age, education, race, and gender profiles in the control group were similar to those in the intervention group, as expected for a randomized experiment. Chi-square independence tests for contingency tables were not significant after multiplicity adjustment. The same was true for the AMT sample.

**Table 1:** Demographic information of the study participants.

Characteristic			AMT^a^, n (%)						Community hospital, n (%)						Overall, n (%)
			Baseline (n=100)		NoteAid (n=100)		Total (n=200)		Baseline (n=85)		NoteAid (n=89)		Total (n=174)		Total (N=374)
**Age**															
	18-21	2 (2.0%)		1 (1.0%)		3 (1.5%)		2 (2.4%)		3 (3.4%)		5 (2.9%)		8 (2.1%)	
	21-34	54 (54.0%)		61 (61.0%)		115 (57.5%)		16 (18.8%)		7 (7.9%)		23 (13.2%)		138 (36.9%)	
	35-44	27 (27.0%)		25 (25.0%)		52 (26.0%)		12 (14.1%)		8 (9.0%)		20 (11.5%)		72 (19.3%)	
	45-54	11 (11.0%)		9 (9.0%)		20 (10.0%)		12 (14.1%)		18 (20.2%)		30 (17.2%)		50 (13.4%)	
	55-64	5 (5.0%)		3 (3.0%)		8 (4.0%)		14 (16.5%)		30 (33.7%)		44 (25.3%)		52 (13.9%)	
	≥65	1 (1.0%)		1 (1.0%)		2 (1.0%)		27 (31.8%)		18 (20.2%)		45 (25.9%)		47 (12.6%)	
	Unknown	0 (0.0%)		0 (0.0%)		0 (0.0%)		2 (2.4%)		5 (5.6%)		7 (4.0%)		7 (1.9%)	
**Education**															
	Less than high school	0 (0.0%)		0 (0.0%)		0 (0.0%)		4 (4.7%)		6 (6.7%)		10 (5.7%)		10 (2.7%)	
	High school	24 (24.0%)		28 (28.0%)		52 (26.0%)		34 (40.0%)		34 (38.2%)		68 (39.1%)		120 (32.1%)	
	Associate’s degree	17 (17.0%)		22 (22.0%)		39 (19.5%)		15 (17.6%)		14 (15.7%)		29 (16.7%)		68 (18.2%)	
	Bachelor’s degree	53 (53.0%)		42 (42.0%)		95 (47.5%)		18 (21.2%)		16 (18%)		34 (19.5%)		129 (34.5%)	
	Master’s degree	6 (6.0%)		8 (8.0%)		14 (7.0%)		12 (14.1%)		14 (15.7%)		26 (14.9%)		40 (10.7%)	
	Unknown	0 (0.0%)		0 (0.0%)		0 (0.0%)		2 (2.4%)		5 (5.6%)		7 (4.0%)		7 (1.9%)	
**Race**															
	African American	8 (8.0%)		9 (9.0%)		17 (8.5%)		3 (3.5%)		2 (2.2%)		5 (2.9%)		22 (5.9%)	
	American Indian	0 (0.0%)		0 (0.0%)		0 (0.0%)		0 (0.0%)		1 (1.1%)		1 (0.6%)		1 (0.3%)	
	Asian	8 (8.0%)		2 (2.0%)		10 (5.0%)		8 (9.4%)		10 (11.2%)		18 (10.3%)		28 (7.5%)	
	Hispanic	15 (15.0%)		6 (6.0%)		21 (10.5%)		11 (12.9%)		14 (15.7%)		25 (14.4%)		46 (12.3%)	
	White	69 (69.0%)		83 (83.0%)		152 (76.0%)		61 (71.8%)		57 (64.0%)		118 (67.8%)		270 (72.2%)	
	Unknown	0 (0.0%)		0 (0.0%)		0 (0.0%)		2 (2.4%)		5 (5.6%)		7 (4.0%)		7 (1.9%)	
**Gender**															
	Female	39 (39.0%)		42 (42.0%)		81 (40.5%)		44 (51.8%)		43 (48.3%)		87 (50.0%)		168 (44.9%)	
	Male	61 (61.0%)		58 (58.0%)		119 (59.5%)		38 (44.7%)		40 (44.9%)		78 (44.8%)		197 (52.7%)	
	Refrain	0 (0.0%)		0 (0.0%)		0 (0.0%)		1 (1.2%)		1 (1.1%)		2 (1.1%)		2 (0.5%)	
	Unknown	0 (0.0%)		0 (0.0%)		0 (0.0%)		2 (2.4%)		5 (5.6%)		7 (4.0%)		7 (1.9%)	

^a^AMT: Amazon Mechanical Turk.

### Effect of the Intervention and Participant Recruitment Source

Table 2 shows the descriptive statistics (proportion correct) of the four groups. Results of the two-way ANOVA are presented in Table 3. Note the CIs in Table 3 are not simultaneous CIs, but one-at-a-time CIs.

The interaction effect in the ANOVA was not significant (*P*=.89), suggesting no evidence that the effect of NoteAid was different across the two participant recruitment sources. Further analyses show that both the main effects of *source* (AMT or community hospital) and of *condition* (baseline or intervention) were significant. Participants who took the ComprehENotes test on AMT on average scored higher than the community hospital participants, and the difference was significant. In addition, participants who had access to NoteAid scored higher than those who did not have access to NoteAid. The difference was again significant.

Our analysis with GLMs yielded similar results. The main effect of *condition* was significant (odds ratio [OR] 1.23, 95% CI 1.10-1.38; *P*<.001), the main effect of *source* was significant (OR 1.33, 95% CI 1.18-1.49; *P*<.001), and the interaction effect between *source* and *condition* was not significant (*P*=.72).

**Table 2:** Summary statistics for the source by condition contingency table in our analysis of variance.

Source	Condition	
	Control^a^	Intervention^a^
AMT^b^	0.756 (0.246), n=100	0.830 (0.201), n=100
Community hospital	0.646 (0.179), n=85	0.727 (0.191), n=89

^a^Data are presented as mean proportion correct (SD), sample size.

^b^AMT: Amazon Mechanical Turk.

**Table 3:** Analysis of variance table.

Variable	df	Sum squares	Mean squares	*F*	Cohen *d* (95% CI)
Source	1	1.06	1.06	24.70^a^	0.52 (0.31 to 0.72)
Condition	1	0.56	0.56	13.06^a^	0.37 (0.17 to 0.58)
Source × condition	1	0.001	0.001	0.02	0.03 (−0.38 to 0.44)
Residuals	370	15.88	0.04	N/A^b^	N/A

^a^*P*<.001.

^b^N/A: not applicable.

### Effects of Demographic Variables

To study the effects of demographic variables, the single case of an American Indian, the two cases where individuals refrained from reporting gender, and the seven cases with missing demographic information were removed from the data set before analyses. To mitigate data sparsity in the contingency table, the category “less than high school” was combined with “high school” in *education*, and the highest and lowest age groups were combined with their adjacent groups. Results from ANOVA and GLMs gave qualitatively the same results. However, ANOVA yielded predicted scores exceeding one and demonstrated clear violation of homoscedasticity. We chose to report results from the GLMs. An outlying residual was identified in the analyses, but its removal yielded similar results.

The only significant effect involving a demographic variable was the interaction between *source* and *education* (χ^2^_3_=16.9, *P*<.001). This shows that the effect of *education* differed across the AMT and community hospital participants. Separate analyses of data from the two groups found that *education* did not have a significant main effect (χ^2^_3_=2.05, *P*=.56) in the AMT group, but had a significant main effect (χ^2^_3_=37.30, *P*<.001) in the community hospital group. Pairwise comparisons revealed that community hospital participants with “high school or lower” education had significantly lower performance than those with a bachelor’s degree and those with a master’s degree. The interaction between *condition* and every demographic variable was not significant, suggesting no evidence of any variations in the effect of NoteAid for people in different demographic categories.

## Discussion

### Principal Results

In this work, we have demonstrated the effectiveness of NoteAid for improving EHR note comprehension in participants recruited from two different sources (a community hospital setting and AMT). For both samples, access to NoteAid significantly improved ComprehENotes scores (RQ1). To the best of our knowledge, NoteAid is the only tool available that has been shown to improve patient health literacy, both in this work and in prior work [25,26]. Samples recruited from these two sources varied, in particular with regard to age and education. AMT participants were younger and more educated than participants recruited from the community hospital. Consistent with prior findings on health literacy and education levels [7], the community hospital participants scored significantly lower than the AMT participants on the ComprehENotes test (RQ2).

Although there were demographic differences between the participant recruitment sources (LGH and AMT), we did not find evidence that the effect of NoteAid was different across the recruitment sources (RQ3). We found that age, race, and gender did not have a significant effect on scores. We did find that the effect of education differed between the two sources (RQ4). For the LGH sample, participants with a high school education or less had significantly lower scores than individuals with a bachelor’s or master’s degree. This result is consistent with existing literature that those with less education are at greater risk for low health literacy [7]. In contrast, no education effect was detected for the online platform AMT participants (RQ5). It is important to include participants recruited from an actual hospital setting to confirm the effectiveness of health literacy tests (eg, ComprehENotes) and tools (eg, NoteAid) across samples.

Results from both LGH and AMT confirmed prior research on the effectiveness of NoteAid for improving participant EHR note comprehension [18,26]. We showed that participants with access to NoteAid achieved higher scores on average than those without access to NoteAid, consistent with both patient self-reporting and empirical analyses in prior work [18,26]. However, this is the first study to test NoteAid empirically in participants recruited from a community hospital setting. While the effectiveness of NoteAid was consistent across our participant recruitment sources, participants from the two sources varied in terms of key characteristics (eg, age and education).

Electronic patient portals are becoming more common, with as many as 44 million patients estimated to have access to their notes [32]. Notes are being made available as part of the OpenNotes initiative [33] and in a variety of other health care settings, from the US Department of Veterans Administration [34,35] to private organizations [36]. As a result, more information about personal health is available to patients. In a recent study, surveyed patients reported that they understood “most or all” of the content in their EHR notes [37]. However, all patients surveyed had previously read at least one of their notes, and most would be considered highly activated patients. Patients who can and do currently view and understand their notes typically have high patient activation [3,38]. They are active in managing their health care, have lower likelihood of outcomes, such as emergency department visits, and are more likely to engage in preventive care [3]. Individuals with low patient activation typically do not understand their role in the health care process and are less likely to participate in tasks associated with health care management (eg, viewing their patient portal) [38]. For those surveyed patients with a high school diploma or lower, self-reported note understanding was significantly lower, consistent with our results [37]. In addition, a common comment from survey respondents was that they had difficulty understanding medical jargon in the note, with respondents requesting access to jargon definitions. Self-reported levels of understanding may not reflect actual understanding, as measured by validated instruments such as ComprehENotes [39,40]. Our results showed significant improvement in comprehension regardless of education level, indicating that NoteAid is an effective intervention.

There are two key challenges for ensuring that patients can realize the benefits associated with accessing their own notes as follows: (1) defining medical jargon so that patients understand the content of their notes, and (2) motivating low activation patients to view their notes and take an active role in managing their health care. The NoteAid tool directly addresses the first challenge, as demonstrated by this work and prior work. As more patients have access to their notes, providing access to definitions at the same time can reduce issues with patients not understanding the content in their newly available notes. It may also indirectly address motivation. If low activation patients struggle with medical jargon, having the jargon automatically defined can reduce the barrier to entry for their participation in care. Implementation of NoteAid directly into EHR software, for example, via an application programming interface (API), would allow for patients to have jargon terms defined within the patient portal itself, without needing to search for definitions outside of the platform. This aligns with a recent call for “easy-to-understand information” as part of a proposal for improving patient portals [41]. Further, NoteAid can be used by anyone who assists in the management of a patient’s care. If a patient chooses to share an EHR note with a family member, the family member can use NoteAid to define medical jargon terms, so that he or she can better understand the note and better assist with the patient’s care. In this case, even patients who themselves might struggle to use NoteAid or read their notes can benefit from the tool.

### Limitations

There are several limitations in this study. First, local recruitment of individuals for our trial had to be halted due to COVID-19. While we were able to gather 174 responses from local participants and identify a significant effect of using NoteAid, a larger sample size would allow for more fine-grained analysis of the results, for example, examination of scores according to various demographic characteristics. While this might be seen as an argument in favor of moving such data collection efforts to crowdsourcing platforms, our results indicate that local population scores are significantly lower than the scores of users on crowdsourcing platforms (RQ2). In addition, the demographic differences between individuals in the local population and individuals on AMT indicate that simply relying on AMT is not sufficient. We confirm prior work demonstrating the effect of NoteAid on EHR note comprehension in AMT workers [26], but go one step further to show that the tool is useful for local patients (a considerably different cohort).

A second limitation is with regard to the NoteAid tool. Certain terms or acronyms in EHR notes can have more than one meaning. The task of correctly identifying the appropriate meaning of an ambiguous term is a well-studied problem in natural language processing called *word sense disambiguation*. While a number of methods have been proposed to handle this problem [42-44], there is active research to improve these models. A NoteAid system in a production environment would need to be able to disambiguate between possible definitions so that a patient would receive the definition for the correct term in his or her own note [25]. In the case of our study, NoteAid definitions were manually inspected and added to the ComprehENotes test as part of our web application implementation. Therefore, we were able to confirm the correct definitions for the terms in our EHR note snippets before running our tasks.

Finally, our local subject group was restricted to diabetes patients and those accompanying them to their appointments. There is a risk that this local setting is too narrow in terms of scope for the results to be more generally applicable. However, the ComprehENotes test is not a test of diabetes EHR note comprehension. The test includes questions from EHR notes related to a number of diseases, such as diabetes, cancer, heart failure, and hypertension, in order to include a wide range of subjects in the assessment [24]. Coupled with the fact that we saw similar results in the AMT participants, we believe that the results could be generalized beyond diabetes patients to a wider patient cohort.

### Conclusion and Future Work

The findings reported here provide evidence of the effectiveness of NoteAid for improving EHR note comprehension across two different participant samples: patients from a community hospital and participants from a popular online crowdsourcing platform. Despite the demographic and education differences between the two samples, NoteAid improved scores on the ComprehENotes test for both, indicating that it is an effective intervention for improving EHR note comprehension. These results support broader use of ComprehENotes as an EHR note comprehension test and NoteAid as an effective tool for improving EHR note understanding.

Future work should explore patient personalization in NoteAid. Providing lay definitions for all medical jargon in a note may lead to information overload for patients with low education and may be unnecessary for patients with high education. To improve comprehension for low health literacy patients, other mediums (eg, short animations and text definitions) may be more effective. Operationally, making NoteAid jargon definitions available for a patient’s own notes via an API would increase the ways in which researchers and EHR vendors can implement jargon definitions so that patients have access to them.

Future work applying the NoteAid tool to other contexts is another interesting direction. While NoteAid has been shown to improve patient EHR note comprehension [25], the methodology of the tool is generalizable. The concept identification-definition linking process can be applied to other texts where complex jargon is common to improve readability and understanding. If a lay-language dictionary is built for a particular domain (eg, legal text), it would be possible to use NoteAid to identify and define those complex terms so that individuals can more easily understand the text.

## Appendix

Multimedia Appendix 1 CONSORT-eHEALTH checklist (V 1.6.1).

## Abbreviations

AMT: Amazon Mechanical Turk
ANOVA: analysis of variance
API: application programming interface
EHR: electronic health record
GLM: generalized linear model
IRB: Institutional Review Board
IRT: item response theory
LGH: Lowell General Hospital
NLP: natural language processing
OR: odds ratio
